# Epigenetic inactivation of the putative DNA/RNA helicase SLFN11 in human cancer confers resistance to platinum drugs

**DOI:** 10.18632/oncotarget.6413

**Published:** 2015-11-27

**Authors:** Vanesa Nogales, William C. Reinhold, Sudhir Varma, Anna Martinez-Cardus, Catia Moutinho, Sebastian Moran, Holger Heyn, Ana Sebio, Agusti Barnadas, Yves Pommier, Manel Esteller

**Affiliations:** ^1^ Cancer Epigenetics and Biology Program (PEBC), Bellvitge Biomedical Research Institute (IDIBELL), Barcelona, Catalonia, Spain; ^2^ Genomics and Bioinformatics Group, Developmental Therapeutics Branch, National Cancer Institute, Bethesda, MD, USA; ^3^ Developmental Therapeutics Branch and Laboratory of Molecular Pharmacology, Center for Cancer Research, National Cancer Institute, Bethesda, MD, USA; ^4^ Department of Medical Oncology, Hospital de la Santa Ceu i Sant Pau, Universitat Autònoma de Barcelona, Barcelona, Catalonia, Spain; ^5^ Department of Physiological Sciences II, School of Medicine, University of Barcelona, Barcelona, Catalonia, Spain; ^6^ Institucio Catalana de Recerca i Estudis Avançats (ICREA), Barcelona, Catalonia, Spain

**Keywords:** SLFN11, CpG island methylation, epigenetics, chemoresistance, DNA-damaging agents

## Abstract

Platinum-derived drugs such as cisplatin and carboplatin are among the most commonly used cancer chemotherapy drugs, but very few specific molecular and cellular markers predicting differential sensitivity to these agents in a given tumor type have been clearly identified. Epigenetic gene silencing is increasingly being recognized as a factor conferring distinct tumoral drug sensitivity, so we have used a comprehensive DNA methylation microarray platform to interrogate the widely characterized NCI60 panel of human cancer cell lines with respect to CpG methylation status and cisplatin/carboplatin sensitivity. Using this approach, we have found promoter CpG island hypermethylation-associated silencing of the putative DNA/RNA helicase Schlafen-11 (SLFN11) to be associated with increased resistance to platinum compounds. We have also experimentally validated these findings *in vitro*. In this setting, we also identified the BRCA1 interacting DHX9 RNA helicase (also known as RHA) as a protein partner for SLFN11, suggesting a mechanistic pathway for the observed chemoresistance effect. Most importantly, we have been able to extend these findings clinically, following the observation that those patients with ovarian and non-small cell lung cancer carrying *SLFN11* hypermethylation had a poor response to both cisplatin and carboplatin treatments. Overall, these results identify *SLFN11* epigenetic inactivation as a predictor of resistance to platinum drugs in human cancer.

## INTRODUCTION

Platinum-derived compounds, such as cisplatin and its second-generation analogue carboplatin, are drugs widely used to treat human malignancies [1]. Either alone or in combination with other antitumor drugs, they have shown to be useful treatments against a broad range of solid cancers, including testicular, ovarian, head and neck, colorectal, bladder and lung cancers [2]. In some cases, such as in testicular cancer, they have even changed the natural history of the disease [3]. Unfortunately, these drugs have several side effects, such as renal impairment, neurotoxicity and ototoxicity, which affect strongly the quality of life of the patients [1, 4]. This fact highlights the importance of discovering biological or cellular markers to predict, within a given tumor, whether a patient's disease will be sensitive or resistant to their antiproliferative effect. However, despite the ubiquity of these agents, we still lack of good biomarkers of platinum agents response that allow us to avoid unnecessary side effects on patients with a resistant malignance. Resistance to platinum agents can be due to several mechanisms [5]. The major cytotoxic mode of action of this kind of drugs is mediated by their interaction with DNA to form DNA adducts which disrupt the structure of the DNA molecule [1]. This alteration of the DNA leads to the activation of DNA damage recognition and repair systems in order to allow cell cycle progression. If the damage cannot be repaired, cell death will be induced through the increase of apoptotic signals [6]. Related to this, resistance to platinum agents can emerge by increasing DNA repair activity or by attenuating DNA damage-mediated apoptotic signals. Thus, one aspect that has recently been garnering interest is the existence of different “repertoires” of DNA repair defects in each patient [7]. In this context, attention has been drawn to BRCA1 for which, in addition to its genetic alterations, the epigenetic inactivation of its expression had been associated to an enhanced platinum response in breast and ovarian tumors [8–10]. Epigenetic inactivation of gene expression by CpG promoter island hypermethylation is a common event in cancer cells [11]. The utility of CpG promoter island hypermethylation events as biomarkers for cancer progression or drugs response have already been demonstrated in several studies [12, 13, 14]. Following this lead, and as reported here, we have adopted a non-biased global genomic approach to identify cancer-specific epigenetic changes that could predict chemosensitivity to platinum-based compounds.

## RESULTS

### DNA methylation analysis of NCI60 cell line panel identifies *SLFN11* CpG promoter island hypermethylation as candidate biomarker of cisplatin and carboplatin resistance

**Figure 1:**
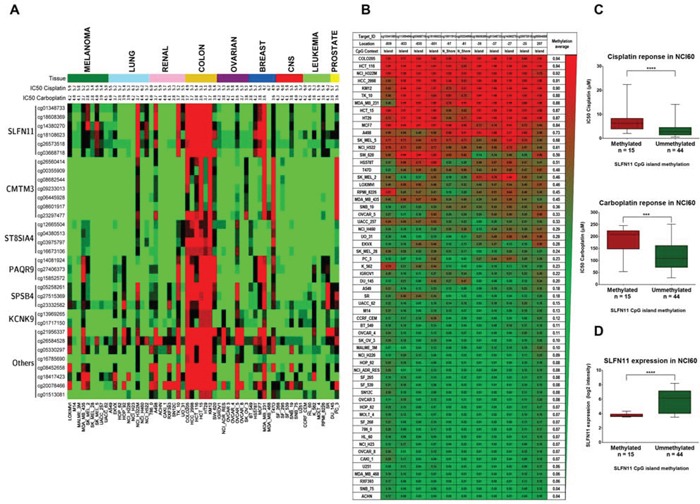
Determination of cisplatin and carboplatin sensitivity in the NCI60 panel of human cancer cell lines with respect to promoter CpG island methylation, analyzed by the 450K DNA methylation microarray **A.** Distribution of promoter CpG island methylation in the NCI60 panel and IC_50_ values for cisplatin and carboplatin (−log [M]). The 33 CpG sites significant for both cisplatin and carboplatin sensitivity are shown. Red, methylated CpG; green, unmethylated CpG. **B.** DNA methylation profile of the CpG island promoter for the SLFN11 gene with respect to DNA methylation microarray values in the NCI60 panel. Single CpG absolute methylation levels (0 – 1) are shown. Red, methylated; green, unmethylated. **C.** Box and whisker plots demonstrating that SLFN11 promoter CpG island hypermethylation is significantly associated with decreased sensitivity to cisplatin and carboplatin in the NCI60 panel. ***p < 0.001: ****p < 0.0001, Student's t test. (C) Box and whisker plots demonstrating SLFN11 promoter CpG island hypermethylation is significantly associated with decreased SLFN11 RNA levels in the NCI60 panel. ****p < 0.0001, Student's *t* test.

To achieve our particular goal, we have interrogated a comprehensive DNA methylation microarray platform [15] for the NCI60 cancer cell line panel in relation to their validated cisplatin and carboplatin sensitivity data [16, 17]. Overall, we analyzed 482,422 CpGs in the 60 cancer cell lines of the NCI60 panel (Figure 1A). The complete DNA methylation data are freely available from the GEO (http://www.ncbi.nlm.nih.gov/geo/query/acc.cgi?token=srydsegkptafnwj&acc=GSE66872) and NCI60 websites. In order to identify strong candidate genes with differential methylation respect to their cisplatin or carboplatin sensitivity, we imposed stringent criteria and only considered those CpG sites harbored in CpG islands located within ± 1,500 bp of the transcription start site of the corresponding gene. These CpG sites were then analyzed along the NCI60 panel by examining the Pearson's correlation coefficients between their methylation values and the cisplatin and carboplatin IC_50_ values obtained from the Developmental Therapeutics Program of the NCI (http://dtp.nci.nih.gov/). Through this analysis we obtained a total of 33 CpG sites which methylation correlated significantly for both cisplatin and carboplatin IC_50_ values (Table 1). Most importantly, we found the highest correlation between methylation and cisplatin and carboplatin resistance in several CpG sites located in the CpG promoter island of Schlafen-11 (*SLFN11*). The correlation between high *SLFN* methylation levels and increased resistance to cisplatin and carboplatin was maintained even if we excluded colorectal (all methylated) and leukemia/CNS (mostly unmethylated) cell lines (Supplementary Figure S1). Given these results, we decided to study the methylation status of the whole CpG promoter island of *SLFN11* and its relation with cisplatin and carboplatin IC_50_ values. *SLFN11* presents a CpG island located around its transcription start site what makes it a candidate gene for hypermethylation-associated inactivation in human cancer (Figure 1B). The DNA methylation microarray approach in the NCI60 panel of cancer cell lines revealed *SLFN11* methylation (higher than 0.5) in 25% (15 of 59) of cell lines, whereas for the remaining 75% (44 of 59) of cell lines the 5′-end CpG island remained unmethylated (lower than 0.5) (Figure 1B). All normal tissues analyzed in our cohort (*n* = 64), counterparts of the NCI60 cancer types, were found to be unmethylated at the *SLFN11* CpG island.

**Table 1:** Common candidate CpGs for Cisplatin and Carboplatin

TargetID	CHR	MAPINFO	UCSC_REFGENE_NAME	UCSC_REFGENE_GROUP	Pearson Cisplatin	Pval Cisplatin	Pearson Carboplatin	Pval Carboplatin
cg01348733	17	33700757	SLFN11;SLFN11;SLFN11; SLFN11;SLFN11	TSS200;TSS200; TSS200;TSS200; TSS200	0.61	1.66E-07	0.55	5.55E-06
cg18608369	17	33700759	SLFN11;SLFN11;SLFN11; SLFN11;SLFN11	TSS200;TSS200; TSS200;TSS200; TSS200	0.61	2.41E-07	0.55	4.36E-06
cg14380270	17	33700747	SLFN11;SLFN11;SLFN11; SLFN11;SLFN11	TSS200;TSS200; TSS200;TSS200; TSS200	0.59	7.52E-07	0.52	2.06E-05
cg18108623	17	33701321	SLFN11;SLFN11;SLFN11; SLFN11;SLFN11	TSS1500;TSS1500; TSS1500;TSS1500; TSS1500	0.59	4.96E-07	0.52	1.97E-05
cg26573518	17	33700745	SLFN11;SLFN11;SLFN11; SLFN11;SLFN11	TSS200;TSS200; TSS200;TSS200; TSS200	0.58	1.03E-06	0.52	2.33E-05
cg26560414	16	66638433	CMTM3;CMTM3;CMTM3; CMTM3;CMTM3	5′UTR;5′UTR;5′UTR; 1stExon;TSS200	0.56	3.11E-06	0.56	3.68E-06
cg00355909	16	66638320	CMTM3;CMTM3;CMTM3; CMTM3; CMTM3;CMTM3;CMTM3	5′UTR;5′UTR;5′UTR; 1stExon;1stExon; 1stExon;TSS1500	0.56	2.54E-06	0.54	8.67E-06
cg03668718	17	33701350	SLFN11;SLFN11;SLFN11; SLFN11;SLFN11	TSS1500;TSS1500; TSS1500;TSS1500; TSS1500	0.56	3.23E-06	0.49	7.12E-05
cg08682544	16	66638438	CMTM3;CMTM3;CMTM3; CMTM3;CMTM3	5′UTR;5′UTR;5′UTR; 1stExon;TSS200	0.55	5.01E-06	0.53	1.10E-05
cg21956337	15	88799707	NTRK3;NTRK3;NTRK3	TSS200;TSS200;TSS200	0.54	9.46E-06	0.57	1.68E-06
cg09233013	16	66638412	CMTM3;CMTM3;CMTM3; CMTM3;CMTM3	5′UTR;5′UTR;5′UTR; 1stExon;TSS200	0.53	1.57E-05	0.53	1.51E-05
cg13969265	8	140716673	KCNK9	TSS1500	0.53	1.13E-05	0.52	1.73E-05
cg14081924	3	142682378	PAQR9	TSS1500	0.53	1.26E-05	0.51	2.73E-05
cg06445928	16	66638407	CMTM3;CMTM3;CMTM3; CMTM3;CMTM3	5′UTR;5′UTR;5′UTR; 1stExon;TSS200	0.52	2.31E-05	0.52	1.93E-05
cg27406373	3	142682291	PAQR9	TSS200	0.52	1.95E-05	0.50	4.62E-05
cg15852572	3	142682288	PAQR9	TSS200	0.52	1.95E-05	0.49	5.95E-05
cg26584528	11	64512032	RASGRP2;RASGRP2; RASGRP2	TSS1500;5′UTR;5′UTR	0.52	1.97E-05	0.49	5.85E-05
cg12665504	5	100239050	ST8SIA4;ST8SIA4	TSS200;TSS200	0.51	2.95E-05	0.56	2.78E-06
cg04380513	5	100238983	ST8SIA4;ST8SIA4;ST8SIA4	TSS200;1stExon;5′UTR	0.51	3.30E-05	0.54	6.54E-06
cg05330297	14	90527606	KCNK13	TSS1500	0.51	2.94E-05	0.54	7.47E-06
cg03975797	5	100238977	ST8SIA4;ST8SIA4;ST8SIA4	TSS200;1stExon;5′UTR	0.51	3.60E-05	0.52	2.22E-05
cg16785690	9	135037323	NTNG2	TSS200	0.50	5.45E-05	0.55	5.15E-06
cg08452658	15	83776271	TM6SF1;TM6SF1	TSS200;TSS200	0.50	3.79E-05	0.51	3.34E-05
cg08601917	16	66638396	CMTM3;CMTM3;CMTM3; CMTM3;CMTM3	5′UTR;5′UTR;5′UTR; 1stExon;TSS200	0.50	5.11E-05	0.51	2.73E-05
cg01717150	8	140716495	KCNK9	TSS1500	0.50	4.58E-05	0.49	6.87E-05
cg18417423	6	80657436	ELOVL4	TSS200	0.50	5.10E-05	0.48	8.51E-05
cg23297477	16	66638293	CMTM3;CMTM3; CMTM3;CMTM3; CMTM3;CMTM3; CMTM3	5′UTR;5′UTR;5′UTR; 1stExon;1stExon; 1stExon;TSS1500	0.49	5.72E-05	0.60	4.84E-07
cg05258261	3	140770608	SPSB4	TSS200	0.49	7.85E-05	0.56	3.66E-06
cg27515369	3	140770599	SPSB4	TSS200	0.49	6.33E-05	0.55	3.98E-06
cg23332582	3	140770308	SPSB4	TSS1500	0.49	6.39E-05	0.52	1.58E-05
cg20078466	7	50344331	IKZF1	TSS200	0.49	8.02E-05	0.50	5.30E-05
cg16673106	5	100239071	ST8SIA4;ST8SIA4	TSS200;TSS200	0.48	8.83E-05	0.54	6.88E-06
cg01513081	6	105584780	BVES;BVES	TSS1500;TSS1500	0.48	8.78E-05	0.48	9.07E-05

Pearson correlation coefficient and *p* value are shown for the methylation value of each CpG respect Cisplatin and Carboplatin IC_50_ values along NCI60 panel.

The classification of cell lines from NCI60 based on the average methylation value of all CpG sites in *SLFN11* CpG promoter island, corroborated the previously obtained association between increased chemoresistance to cisplatin and carboplatin (Figure 1C). Thus, *SLFN11* promoter methylated cell lines group showed cisplatin and carboplatin IC_50_ average values significantly higher than the *SLFN11* unmethylated cell lines group. In addition, by using the available data of *SLFN11* gene expression [18], we found a significant association between *SLFN11* CpG island methylation with diminished *SLFN11* RNA levels (Figure 1D). In this regard, the expression levels of SLFN11 correlate with cisplatin and carboplatin drug sensitivity (18) and we have confirmed these data running the CellMiner Analysis Tool (http://discovery.nci.nih.gov/cellminer/) (Supplementary Figure S2). Importantly, using a tridimentional dot-plot distribution, we found a significant common correlation in the NCI-60 cell lines between the three parameters: IC50 values for sensitivity to platinum drugs, SLFN11 expression and SLFN11 promoter methylation (Supplementary Figure S2). Thus, the candidate DNA methylation-associated silencing of *SLFN11* as a predictor of chemoresistance to platinum agents in cancer became our main focus of interest.

### Hypermethylation of *SLFN11* CpG promoter island is associated with a inactivation of *SLFN11* gene expression in cancer cells

**Figure 2:**
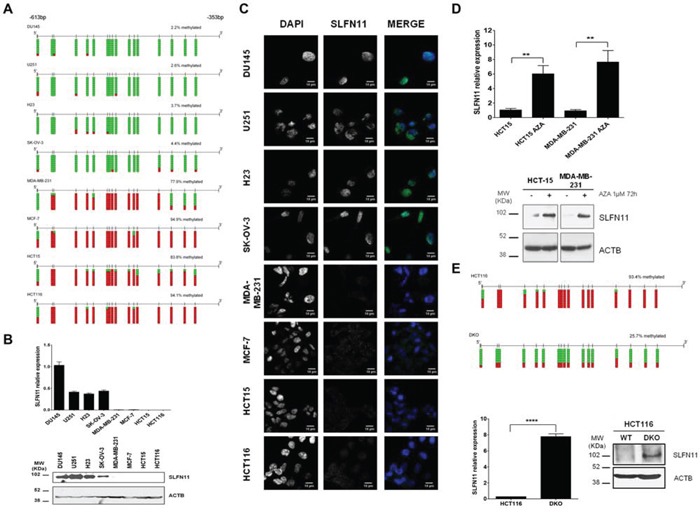
DNA methylation-associated transcriptional silencing of SLFN11 **A.** Bisulfite genomic sequencing of SLFN11 promoter CpG island. CpG dinucleotides are represented as short vertical lines. At least eight single clones are shown for each sample. Presence of a methylated or unmethylated cytosine is indicated by a red or green square, respectively. MDA-MB-231, MCF-7, HCT-15, and HCT-116 show dense CpG island methylation. **B.** Expression levels of the SLFN11 transcript and protein determined by real-time reverse transcription-PCR and western blot, respectively. **C.** SLFN11 (green label) and DAPI (blue label) immunofluorescence in the studied cancer cell lines. **D.** The expression of SLFN11 RNA transcript and protein was restored in the methylated HCT15 and MDA-MB-231 cells by treatment with the demethylating drug 5-aza-2-deoxycytidine (AZA). **E.** Genetic disruption of the two major DNA methyltransferases DNMT1 and DNMT3B (in DKO cells) also restored SLFN11 RNA and protein expression in HCT-116 cells. Data are summarized as the mean ± s.e.m. of three biological replicates. ***p < 0.001,

Having observed the mentioned *SLFN11* promoter CpG island hypermethylation profiles, we assessed in greater detail their association with the putative transcriptional inactivation of the *SLFN11* gene at the RNA and protein levels. To this end, we first performed bisulfite genomic sequencing of multiple clones in eight selected cancer cell lines from different tissue types with different cisplatin and carboplatin sensitivities that confirmed the DNA methylation patterns obtained by the microarray approach (Figure 2A). The cancer cell lines HCT-116, HCT-15, MDA-MB-231, and MCF7, hypermethylated at the *SLFN11* CpG island, had minimal expression of the *SLFN11* RNA transcript, as determined by quantitative reverse transcription-PCR (Figure 2B), and protein, as assessed by western blot (Figure 2B) and immunofluorescence (Figure 2C). By contrast, cancer cell lines unmethylated at the *SLFN11* promoter (U251, NCI-H23, DU145 and SK-OV-3) expressed highly detectable *SLFN11* RNA transcript (Figure 2B) and protein levels (Figure 2B and Figure 2C). We established a further link between *SLFN11* CpG island hypermethylation and its gene silencing by treating the HCT-15 and MDA-MB-231 cell lines with a DNA-demethylating agent. Treatment of these *SLFN11* methylated cell lines with 5-aza-2-deoxycytidine restored *SLFN11* expression at the RNA and protein levels (Figure 2D). These results were confirmed in the isogenic HCT-116 cell line, in which the two major DNA methyltransferases, DNMT1 and DNMT3B, had been genetically disrupted (DKO) [19]. We observed that the *SLFN11* CpG island was unmethylated in double knockout (DKO) cells and, most importantly, that *SLFN11* transcription was restored as showed the quantitative reverse transcription-PCR assay (Figure 2E). Western blot analysis confirmed the absence of SLFN11 protein expression in HCT-116 cells and its recovery in DKO cells (Figure 2E). Importantly, we found that pre-treatment of the SLFN11 hypermethylated cell line MDA-MB-231 with the inhibitor of DNA methylation 5-azacytidine followed by the addition of cisplatin or carboplatin increased the sensitivity to these compounds (Supplementary Figure S3). In a similar manner, DKO cells (undergoing the described demethylation-associated reactivation of SLFN11) were also more sensitive to cisplatin or carboplatin than the parental HCT-116 cell line (Supplementary Figure S3).

### *In vitro* silencing of *SLFN11* gene expression increases resistance to cisplatin and carboplatin treatments

**Figure 3:**
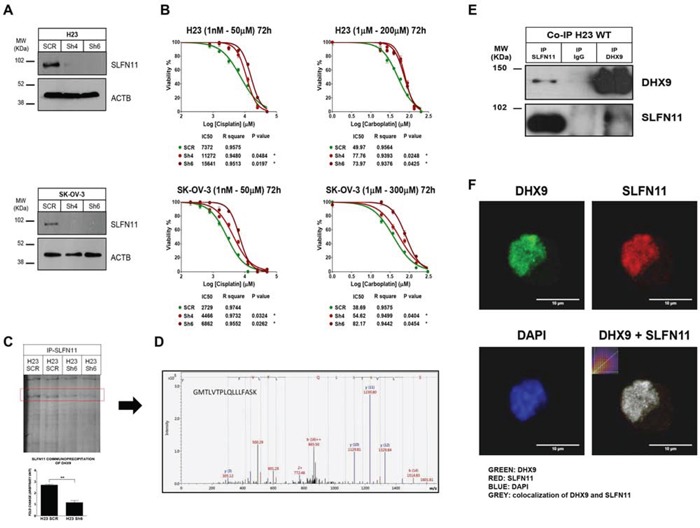
Impact of SLFN11 in cisplatin/carboplatin chemoresistance *in vitro* and the search for a protein partner **A.** Western blot showing the *in vitro* depletion by the short hairpin RNA approach in SK-OV-3 (ovarian) and NCI-H23 (lung) cancer cell lines of the SLFN11 protein. **B.** Cell viability determined by the 3-(4,5-dimethyl-2-thiazolyl)-2,5-diphenyl-2H-tetrazolium bromide (MTT) assay following exposure to cisplatin and carboplatin. External intervention by shRNA-mediated depletion gives rise to enhanced resistance to both drugs. The corresponding half-maximal inhibitory concentration (IC_50_) values are also shown. **C.** Gel analysis comparing the SLFN11 protein immunoprecipitates obtained in NCI-H23 cells from the shRNA scramble cells with the SLFN11-shRNA-depleted cells, which reveals the marked reduction of a band, which was isolated for further characterization. **D.** Mass spectrometry (MS) analysis of diferential immunoprecipitates for SLFN11 interactors. The MS/MS fragmentation ions of the last peptide are shown in blue and red corresponding respectively to y and b ions. **E.** Coimmunoprecipitation experiments confirmed the interaction of SLFN11 and DHX9. Immunoprecipitation and western blot were performed using anti-SLFN11 and anti-DHX9 antibodies. Normal mouse IgG was used as a negative control. **F.** Examples of multi-color immunofluorescence images show colocalization of the SLFN11 (red labeling) and DHX9 (green labeling) proteins in the nucleus of NCI-H23 cells.

Having shown the epigenetic silencing of *SLFN11* gene expression by DNA methylation of its CpG promoter island, we next sought to demonstrate that this *SLFN11* epigenetic inactivation functionally contributed to platinum resistance. With that end, we decided to downregulate the expression of *SLFN11* by the short hairpin RNA approach in *SLFN11*-expressing and unmethylated SK-OV-3 and NCI-H23 cells. These cell lines represent two tumor types commonly treated with platinum-based chemotherapy, ovarian and lung cancer, respectively. Two Sh- sequences targeting different regions of *SLFN11* RNA transcript were use to assess the relation between *SLFN11* silencing and platinum resistance. For both shRNA against SLFN11 a notable reduction on SLFN11 protein levels was observed compare to shRNA-scramble cells (Figure 3A). IC_50_ values to cisplatin and carboplatin treatment were then determined for all constructions by MTT assay. Under this shRNA-mediated downregulation of *SLFN11* both cell lines showed statistically significantly increased IC_50_ values for platinum treatments than the shRNA-scramble cells (Figure 3B). Conversely, upon efficient recovery of SLFN11 expression by transfection of the full-length SLFN11 protein in the hypermethylated and silenced HCT-15 and MCF7 cancer cell lines, we observed a significant increase in the sensitivity to cisplatin and carboplatin (Supplementary Figure S4).

### SLFN11 co-immunoprecipites with the BRCA1-interactor DHX9

Although some data have been reported about the putative function of SLFN11 related with immune system response [20], very little is known about the role of SLFN11 in other contexts such as cell response to platinum-induced DNA damage. To gain further knowledge of SLFN11 activity, we searched for protein partners of SLFN11 by combining immunoprecipitation and mass spectrometry (MS). We compared SLFN11 immunoprecipitates obtained from NCI-H23 cells (*SLFN11* unmethylated and expressing the gene) to SLFN11 shRNA-mediated donwregulation and in shRNA-scramble cells (Figure 3A). We observed in the gel lanes of the *SLFN11* downregulated shRNA cells' immunoprecipitates that a particular band was significantly diminished relative to the shRNA-scramble cells (Figure 3C). This band was isolated for further characterization by MS. Mass spectrometry (MS) analysis identified the isolated band as the protein DEAH (Asp-Glu-Ala-His) box helicase 9 (DHX9) with a Mascot Protein score of 83.9, thought the identification of two peptides: DINTDFLLVVLR (score 56.4) and GMTLVTPLQLLLFASK (score 61.4) (Figure 3D). DHX9 is also known as RNA helicase A (RHA) which has been related to DNA damage repair and stability, and characterized as interactor of BRCA1 in this context [21, 22, 23]. Direct binding of SLFN11 to DHX9 was confirmed by co-immunoprecipitation and western-blot using specific antibodies against DHX9 and SLFN11 in NCI-H23, DU145 and U251 cells (Figure 3E and Supplementary Figure S5). We also performed immunefluorescence assays in NCI-H23 cells demonstrating at least partial colocalization of the SLFN11 and DHX9 proteins in the cell nucleus (Figure 3F).

### *SLFN11* CpG promoter island hypermethylation correlates with worse overcome in ovarian or lung cancer patients undergoing treatment with platinum-derived drugs

**Figure 4:**
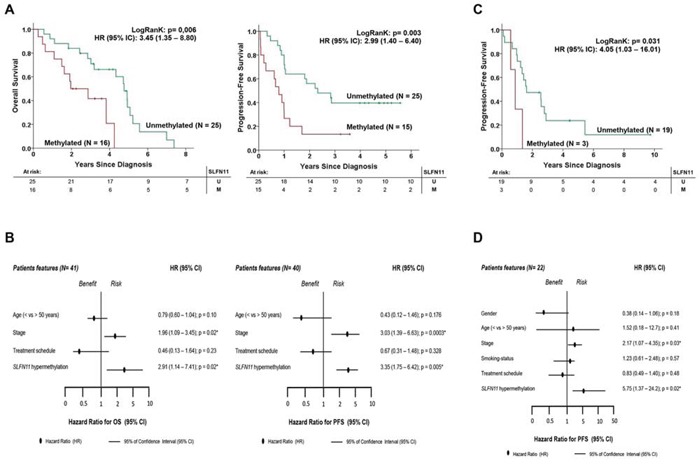
SLFN11 CpG island hypermethylation is an independent factor that is prognostic of poor clinical outcome in ovarian and non-small cell lung cancer patients treated with platinum-derived drugs **A.** Kaplan-Meier analysis of overall survival (OS) and progression-free survival (PFS) in the ovarian cancer clinical cohort with respect to SLFN11 methylation status. The statistical significance of the log-rank test is shown. Results of the univariate Cox regression analysis are represented by the hazard ratio (HR) and 95% confidence interval (95% CI). The number of cases (n) and the mean time to progression/survival in years (y) is indicated for each group. **B.** Forest plot of multivariate Cox regression, taking into account different clinical features of the validation cohort. Parameters with an associated value of *p* < 0.05 were considered as independent prognostic factors. HR associated with PFS and OS in the ovarian cancer cohort. **C.** Kaplan-Meier analysis of PFS among the non-small cell lung cancer clinical cohort with respect to SLFN11 methylation status. The significance of the log-rank test is shown. Results of the univariate Cox regression analysis are represented by the HR and 95% CI. The number of cases (n) and the mean time to progression/survival in years (y) are indicated for each group. **D.** Forest plot of multivariate Cox regression analysis, taking into account a range of clinical features of the validation cohort. Parameters with an associated value of *p* < 0.05 were considered to be independent prognostic factors. HR associated with PFS in the non-small cell lung cancer cohort.

Finally, given our *in vitro* findings that cancer cells with *SLFN11* methylation-associated silencing are resistant to cisplatin and carboplatin, we wondered whether the same effect could be observed in clinical samples. To address this question, we studied two cohorts of patients with primary ovarian and non-small cell lung tumors, both of which are human malignancies in which platinum-derived treatments are commonly used [1, 2]. The study of a first clinical cohort of 41 cases of papillary serous ovarian cancer (Table 2), all of whom were treated with cisplatin or carboplatin, showed *SLFN11* methylation in 39% (*n* = 16 of 41) of the patients analyzed by methylation-specific PCR. Importantly, we observed that *SLFN11* hypermethylation was significantly associated with shorter overall survival (OS) (log-rank test, *P* = 0.006; HR = 3.45; 95% confidence interval [CI] = 1.35 – 8.80) (Figure 4A). For the 40 patients for whom progression-free survival (PFS) information was available, *SLFN11* hypermethylation was also significantly associated with shorter PFS (log-rank test, *P* = 0.003; HR = 2.99; 95% CI = 1.40 – 6.40) (Figure 4A). According to the Cox regression multivariate test, *SLFN11* was an independent prognostic factor of OS (log-rank test, *P* = 0.02; HR = 2.91; 95% CI = 1.14 – 7.41) and PFS (log-rank test, *P* = 0.005; HR = 3.35; 95% CI = 1.75 – 6.42) prognostic factor (Figure 4B). A similar pattern was observed when we studied a clinical cohort of non-small cell lung cancer adenocarcinomas (n = 22) who received platinum-based chemotherapy (Table 3). The presence of *SLFN11* hypermethylation, detected by the DNA methylation microarray method, was found in 13.6% (n = 3 of 22) of the cases. Although OS information was not available for these patients, we found that *SLFN11* CpG island hypermethylation was significantly associated with shorter PFS (log-rank test, P = 0.031; HR = 4.05; 95% CI = 1.03 – 16.01) (Figure 4C). The Cox multivariate regression model showed that *SLFN11* also was an independent prognostic factor of PFS (log-rank test, P = 0.02; HR = 5.75; 95% CI = 1.37 – 24.2) (Figure 4D). Thus, the clinical results resemble those derived from the aforementioned cell cultures, which suggest increased chemoresistance of *SLFN11* hypermethylated tumors to platinum-derived treatments.

**Table 2:** Clinical features of ovarian cancer cohort

*N* = 41	*N*	%	SLFN11				Methylation Status (U/M)	
			Unmethylated (U)		Methylated (M)		OR (95% CI)	P*
			*N*	%	*N*	%		
**Age**								
<50	5	12%	2	40%	3	60%	1 (reference)	-
>50	21	51%	9	43%	12	57%	1.33 (0.16 - 11.3)	n.s
Unknown	15	37%	14	93%	1	7%	-	-
**Stage**								
I	5	12%	4	80%	1	20%	1 (reference)	-
II	3	7%	3	100%	0	0%	0.50 (0.25 - 1.01)	n.s
III	26	64%	14	54%	12	46%	0.88 (0.72 - 1.08)	n.s
IV	7	17%	4	57%	3	43%	0.74 (0.33 - 1.67)	n.s
**Histology type**								
Papillary serous	41	100%	25	61%	16	39%	-	-
**Chemotherapy schedule**								
Platinum-based schedule	15	37%	14	93%	1	7%	1 (reference)	-
Carboplatin + Taxol	26	63%	11	42%	15	58%	0.47 (0.30 - 0.74)	0.002
**Relapse / Progression**								
No	12	29%	10	83%	2	17%	1 (reference)	-
Yes	28	68%	15	54%	13	46%	2.79 (0.74 - 10.5)	n.s
Unknown	1	3%	0	0%	1	100%	-	-
**Survival**								
Alive	12	30%	7	58%	5	42%	1 (reference)	-
Exitus	29	70%	18	62%	11	38%	1.05 (0.69 - 1.58)	n.s

* *P*-value was assessed according Chi-Square test; *p* < 0.05 as statistical significant.

*SLFN11* methylation status is shown along to the different clinical features of patients.

**Table 3:** Clinical features of non-small cell lung cancer adenocarcinoma cohort

*N* = 22	*N*	%	SLFN11				Methylation Status (U/M)	
			Unmethylated (U)		Methylated (M)		OR (95% CI)	P*
			*N*	%	*N*	%		
**Gender**								
Male	10	45%	8	80%	2	20%	1 (reference)	-
Female	12	55%	11	92%	1	8%	0.36 (0.03 – 4.74)	n.s
**Age**								
<50	2	9%	2	100%	0	0%	1 (reference)	-
>50	20	91%	17	85%	3	15%	0.89 (0.77 – 1.04)	n.s
**Stage**								
I	6	27%	5	83%	1	17%	1 (reference)	-
II	7	32%	5	71%	2	29%	1.50 (0.27 – 8.34)	n.s
III	9	41%	9	100%	0	0%	0.35 (0.17 – 0.72)	n.s
IV	0	0%	-	-	-	-	-	-
**Smoking-status**								
No	5	23%	4	80%	1	20%	1 (reference)	-
Former	12	54%	11	92%	1	8%	0.53 (0.11 – 2.69)	n.s
Yes	5	23%	4	80%	1	20%	1.00 (0.21 – 4.71)	n.s
**Treatment schedule**								
RT plus Platinum-based CT	8	36%	6	75%	2	25%	1 (reference)	-
Platinum plus Taxane	5	23%	4	80%	1	20%	0.90 (0.35 – 2.32)	n.s
Platinum-based CT	9	41%	9	100%	0	0%	0.40 (0.21 - 0.74)	n.s
**Relapse**								
No	4	21%	4	100%	0	0%	1 (reference)	-
Yes	18	79%	15	83%	3	17%	0.79 (0.63 - 1.01)	n.s

* *P*-value was assessed according Chi-Square test; *p* < 0.05 as statistical significant.

Abbreviations: RT = Radiotherapy; CT = Chemotherapy; n.s = not significant

*SLFN11* methylation status is shown along to the different clinical features of patients.

## DISCUSSION

Herein, we have analyzed the DNA methylome of the well characterized cancer cell line panel NCI60 by using the 450K microarray from illumina, and we have related the obtained DNA methylation patterns with the available data of cisplatin and carboplatin response from the Developmental Therapeutics Program of the NCI (http://dtp.nci.nih.gov/). Using this approach we have been able to identify *SLFN11* CpG promoter island hypermethylation as a predictive biomarker of platinum resistance.

*SLFN11* expression has been previously related with cells sensitivity to several DNA damage drugs as topotecan, cisplatin and irinotecan [18, 24, 25]. SLFN11 belongs to the Schlafen protein family, which has been implicated in the regulation of important biological functions, such as control of cell proliferation and induction of immune responses [26], regulation of viral replication [20], and, for cancer, in sensitizing cancer cells to DNA damaging agents [18, 24, 25]. The SLNF11 C-terminal is constituted by a DNA/RNA helicase-like motif that has not been functionally characterized yet. This kind of motif has been described to participate in essentials roles of the DNA damage cell response [27]. Previous proteomic studies pointed SLFN11 as a putative interactor of several key proteins of the DNA damage cell response system, such as replication proteins RPA1, RPA2 and RPA3 or BRCA1-Associeted Ring Domain protein (BARD1) [28, 29]. Herein, our mass spectrometry and co-immunoprecipitation analyses indicate that the BRCA1-interactor DHX9 as a partner of SLFN11. DHX9 is a member of the DEAH-containing family of RNA helicases with an enzyme activity that catalyzes the ATP-dependent unwinding of double-stranded RNA and DNA-RNA complexes [30, 31]. Most importantly, DHX9 has been implicated in genome maintenance processes [21, 22]. DHX9 has several interacting partners that are directly involved in DNA repair, the most prominent being the tumor suppressor BRCA1 [32]. Interestingly, epigenetic defects in BRCA1 have been associated with increased sensitivity to platinum-derived drugs in human cancer [8–10], whilst an impairment of at least one BRCA1-interactor protein (SRBC) has been related to increased resistance to these compounds [13]. Thus, it is reasonable to speculate that *SLFN11* epigenetic silencing compromises the correct partnership of DHX9 and BRCA1, and then it alters the correct function of the DNA damage response system, causing a shift in the platinum-associated chemosensitivity of the affected cancer cells.

Finally, in our study we also show that this epigenetic molecular alteration can be used as a response predictive biomarker to platinum-based therapy in ovarian and lung human cancer. Since all tumor samples in our studies were collected before chemotherapy we presume that the observed hypermethylation of *SLFN11* in the analyzed tumors confers a growth advantage in these cells and it is also a biomarker of primary resistance to the mentioned drugs. Future studies on tumor samples before and after treatment should be performed to determine whether promote CpG island hypermethylation of *SLFN11* could be a subject of cellular selection generating secondary resistance and, if so, whether it could be used as a biomarker for the selection of the most useful chemotherapy regimen.

## MATERIALS AND METHODS

### Human cancer cells and tissues

All cell lines were obtained from the American Type Culture Collection (Manassas, VA). All cell lines were cultured at 37°C in an atmosphere of 5% (v/v) carbon dioxide. HCT-116, HCT15, MCF-7, MDA-MB-231 and 293T cell lines were cultured in Dulbecco's Modified Eagle's Medium (DMEM) while OVCAR-8, H23, DU145 and U251 were cultured in Roswell Park Memorial Institute medium (RPMI1640). The medium was always supplemented with 10% (w/v) fetal bovine serum, 100U penicillin, and 100ug/L streptomycin (Invitrogen, Carlsbad, CA). Cells were authenticated by STR profiling and tested for the absence of mycoplasma. Primary ovarian and non-small cell lung cancer samples were obtained from the Hospital de la Santa Ceu i Sant Pau and the Cancer Epigenetics and Biology Program Biobanks. Progression free survival (PFS) and overall survival (OS) was determined by using Kaplan-Meier plots and Log-Rank test. Statistical analysis was performed by using SPSS for Windows and *p*-values under 0.05 were considered statistic significant.

### DNA methylation arrays

Whole-genome DNA methylation was analyzed in the sixty cell lines of NCI60 panel using the Illumina Infinium HumanMethylation450Beadchips. DNA was extracted from cell lines and tissues by the phenol:chloroform method. All DNA samples were assessed for integrity, quantity and purity by electrophoresis in a 1.3% agarose gel, picogreen quantification, and nanodrop measurement. All samples were randomly distributed into 96-well plates. Bisulfite conversion of 500 ng of genomic DNA was performed using an EZ DNA methylation kit (Zymo Research) following the manufacturer's instructions. 200 ng of bisulfite converted DNA were used for hybridization on the HumanMethylation450 BeadChip (Illumina). Briefly, samples were whole-genome amplified followed by enzymatic end-point fragmentation, precipitation and resuspension. The resuspended samples were hybridized onto the beadchip for 16 h at 48°C and washed. Single nucleotide extension with labeled dideoxy-nucleotides was performed and repeated rounds of staining were carried out with a combination of labeled antibodies differentiating between biotin and DNP. DNP and biotin staining, hybridization, target removal, extension, bisulfite conversion G/T mismatch, negative and non-polymorphic control probe intensities were inspected as recommended by Illumina. Raw fluorescence intensity values were normalized with Illumina Genome Studio software (V2011.1) using “control normalization” with background correction. Normalized intensities were then used to calculate DNA methylation levels (beta values). Likewise, data points with statistically low power (as reported by detection values of *p* > 0.01) were designated as NA and excluded from the analysis. Genotyping probes present on the chip as well as DNA methylation probes overlapping with known single-nucleotide polymorphisms (SNPs) were also removed. Probes were considered to be in a promoter CpG island if they were located within a CpG island (UCSC database) and less than 1,500 bp away from a transcription start site.

### Bisulfite sequencing

Genomic DNA was converted using an EZ DNA Methylation Gold kit (Zymo Research, Orange, CA, USA). A specific region of the promoter island was amplified by PCR and cloned in competent bacteria. A minimum of eight single clones were interrogated for each sample and the methylation frequency was calculated in each case.

### Expression analysis

For qRT–PCR experiments, total RNA was extracted using Trizol^®^ reagent and retrotranscribed using the ThermoScript™ RT–PCR System (Invitrogen). The reaction was carried out following the methods for use of SYBR Green (Applied Biosystems), and *HPRT* were used as housekeeping gene to enable normalization. Reactivation treatments with the demethylating agent 5-aza-2′-deoxycytidine (AZA; Sigma) were performed at 1 μM for 72 h. For immunoblotting assays, total protein was extracted using RIPA (50 mM Tris pH 7.5, 150 mM NaCl, 1 mM EDTA and EGTA, 1% NP40, 0.5% of sodium deoxycholate, 0.1% of SDS, and protease and phosphatase inhibitors from Roche), and specific antibodies against target proteins are listed in the enclosed table.

### Short hairpin interference and ectopic expression assays

Six different short hairpin RNAs (shRNAs) were designed over the *SLFN11* mRNA to target ovarian and lung SLFN11-expressing cells. A shRNA against the MSS2 yeast protein (not present in mammals) was used as scrambled. All annealed shRNA oligos were ligated into pLVX-shRNA2 plasmid, purchased from Clontech, using BamH1 and EcoR1 restriction sites. 10 μg of each shRNA-encoding plasmid were mixed with 7.5 μg of ps-PAX2 and 2.5 μg of PMD2.G plasmid in 1 ml JetPRIME buffer and 50ul of JetPRIME (Polyplus-transfection S.A., Illkirch, France). After 10 min of RT incubation, the transfection mix was added drop-wise to a 10 cm dish containing 10 ml of DMEM and 293T cells at 80% confluence. After 48 h, viral supernatant was recovered, 0.45-μm filtered and added to six-well plates containing the host cells at 80% confluence. After 48–72 h, cells were checked for infection efficiency. For ectopic expression assays, normal colon RNA was retrotrascribed and used to amplify SLFN11 transcript. Specific adapter primers were used to add restrition sites for NotI and XhoI to 5′ and 3′ ends of SLFN11 amplification. PCR product and pcDNA4/TO expresion vector were digested with fast digest XhoI and NotI restriction enzymes (New England) and ligated by using T4 enzyme. Competent bacteria were transformed with the pcDNA4/TO-SLFN11 vector, grown and processed by maxiprep technique. SLFN11 total sequence carried in the obtained vector was verified by sequencing PCR. HCT-15 and MCF-7 cells in exponential growth were electroporated to introduce pcDNA4/TO empty vector or pcDNA4/TO carrying SLFN11 cds.

### Mass spectrometry analysis

1 mg of nuclear protein extract was recovered in 1 ml of RIPA (50 mM Tris pH 7.5, 150 mM NaCl, 1 mM EDTA and EGTA, 1% NP40, 0.5% of sodium deoxycholate, 0.1% of SDS, and protease and phosphatase inhibitors from Roche) and putted on ice. After cleaning the membrane residues, overnight pre-cleaning was done with magnetic beads (Dynabeads^®^ M-280 Sheep Anti-Mouse IgG, Invitrogen) at 4°C. 10 μg of anti-SLFN11 antibody from Santacruz (sc-374339) was incubated with 30 μl of magnetic beads overnight at 4°C in 500ul of PBS. After that, beads were removed from the protein solution and magnetic beads with antibody were recovered. Beads were then cleaned three times with mild wash buffer (PBS plus 0.1% of NP40). Target proteins were eluted using Laemmli buffer at 90°C for 10 min and eluted proteins were separated from beads. Samples were loaded and run on a 7.5% polyacrylamide gel. Bands were identified staining the gel with silver overnight with Silver Quest kit (Invitrogen), following the manufacturers protocol. Single bands were excised and sent to the proteomics service of IDIBELL (Spain). Mass spectrometry was performed in a NanoAcquity (Waters) HPLC coupled to an LTQ OrbitrapVelos mass spectrometer (Thermo Scientific). Data analysis was carried out in the LTQ OrbitrapVelos. Peptide masses were measured in the Orbitrap at a resolution of 60,000 (m/z: 300 - 1700). The ten most abundant peptides (minimum intensity of 500 counts) were selected from each MS scan and fragmented using CID (38% normalized collision energy) in the linear ion trap with helium as the collision gas. All results were filtered so only proteins identified with high confidence peptides (FDR ≤ 0.01) and with at least two peptides were included. Finally, results were filtered by *Homo sapiens*.

### Mass spectrometry validation

For coimmunoprecipitation (CoIP), H23 wild type cells were grown at 70% confluence. Nuclear extract was prepared with RIPA. Samples were pre-cleared using Dynabeads^®^ M-280 Sheep Anti-Mouse IgG (Invitrogen), overnight at 4°C in rotation. Antibodies against SLFN11 (sc-374339, santacruz) and against RHA (sc-137198, santacruz) were pre-incubated with beads for two hours at 4°C in rotation. Samples were cleared of beads, and beads with the antibodies attached were recovered. Samples were incubated with their respective antibodies at 4°C in rotation for 2–4 h. After that, beads were washed three times with mild wash buffer (PBS plus 0.1% of NP40). Target proteins were then eluted using Laemmli buffer at 90°C for 10 min and elution proteins were separated from beads. To avoid cross-reactivity and unspecific band detection, mouse TrueBlot HRP secondary antibody was used. As a CoIP negative control we used normal mouse Ig from Millipore (12–371) or Anti-Nucleolin (sc-8031, Santacruz).

### Colocalization assay

For colocalization analysis, the cells were cultured directly on polilysinated coverslips and fixed with 4% paraformaldehyde for 10 minutes. Cells were permeabilized with 0.1% Triton X-100 for 10 minutes and blocked with 2% blocking reagent (Roche) for one hour. Immunistaining with SLFN11 (1:300) (sc-136890, Santacruz) and RHA (1:500) (sc-137198, santacruz) was performed for 16 h at 4°C. Anti-mouse Alexa488 was used as secondary antibody for RHA detection and Anti-goat Alexa555 for detection of SLFN11. Multi-color immunofluorescence images were capture and the images obtained analyzed with the plugin Intensity Correlation Analysis (Image J) to calculate the Manders'R overlap colocalization coefficient.

### Cell viability assays

Cells were seeded in either 96-well microplates at ∼15% confluence in medium with 10% FBS and penicillin/streptomycin. The optimal cell number for each was determined to ensure that each one was in growth phase at the end of the assay. After overnight incubation, cells were treated with 5–8 concentrations of cisplatin 1mg/ml (Pfizer) or carboplatin 10mg/ml (Teva) and then returned to the incubator for assay after 72 h. Cell viability was determined by the 3-(4, 5-dimethy-2-thiazolyl)-2, 5-diphenyl-2H-tetrazolium bromide (MTT) assay. MTT was added at final concentration of 0.1%. After 3 hours of incubation (37°C; 5% carbon dioxide), the MTT metabolic product formazan was dissolved in dimethyl sulfoxide (DMSO), and absorbance was measured at 570nm. Prism Software (La Jolla, CA) was used to calculate the drug half-maximum inhibitory concentration (IC_50_).

## SUPPLEMENTARY FIGURES


