# Supplementary material for: Epigenetic inactivation of the putative DNA/RNA helicase SLFN11 in human cancer confers resistance to platinum drugs
Source: Oncotarget. 2015 Nov 27;7(3):3084–97. doi: 10.18632/oncotarget.6413 (PMC4823092; doi:10.18632/oncotarget.6413)
Supplement: Supplementary file 1 [file oncotarget-07-3084-s001.pdf]

SUPPLEMENTARY FIGURES

A

Parameter	IC50 Cisplatinum (µM)	IC50 Carboplatinum (µM)
Number of cell lines (All cell lines included)	60	59
Pearson r	0.6042	0.6018
95% confidence interval	0.4137 to 0.7440	0.4087 to 0.7434
P value (one-tailed)	< 0.0001	< 0.0001
P value summary	****	****
Is the correlation significant? (alpha=0.05)	Yes	Yes
R square	0.3651	0.3622

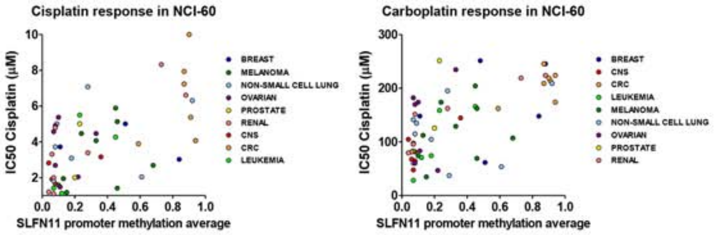

B

Parameter	IC50 Cisplatinum (µM)	IC50 Carboplatinum (µM)
Number of cell lines (CRC, CNS and leukemia cell lines excluded)	41	40
Pearson r	0.5219	0.4072
95% confidence interval	0.2552 to 0.7148	0.1095 to 0.6378
P value (one-tailed)	0.0002	0.0046
P value summary	***	**
Is the correlation significant? (alpha=0.05)	Yes	Yes
R square	0.2724	0.1658

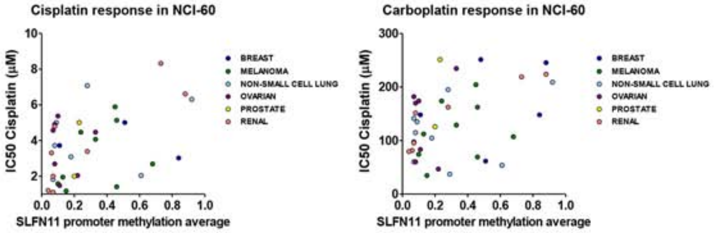

Supplementary Figure S1: Correlational study between SLFN11 promoter methylation status and IC50 values for cisplatin and carboplatin in the NCI-60 panel. A. Pearson correlation analysis for platinum drugs IC_{50} values and promoter methylation average of SLFN11 considering all cell lines in NCI-60 panel. **B.** Pearson correlation analysis for platinum drugs IC_{50} values and promoter methylation average of SLFN11 excluding tissue types homogenously methylated (CRC) and mostly unmethylated (CNS and leukemia) in order to discard a tissue-specific effect. CRC: colorectal cancer. CNS: central nervous system.

	Pearson correlation Coefficient		p-values	
	SLFN11 Methylation	SLFN11 Expression	SLFN11 Methylation	SLFN11 Expression
SLFN11.Methylation	-	-0.66	-	1.15E-08
SLFN11.Expression	-0.66	-	1.15E-08	-
IC50.Cisplatin	0.56	-0.63	3.23E-06	6.76E-08
IC50.Carboblatin	0.51	-0.54	3.07E-05	8.63E-06

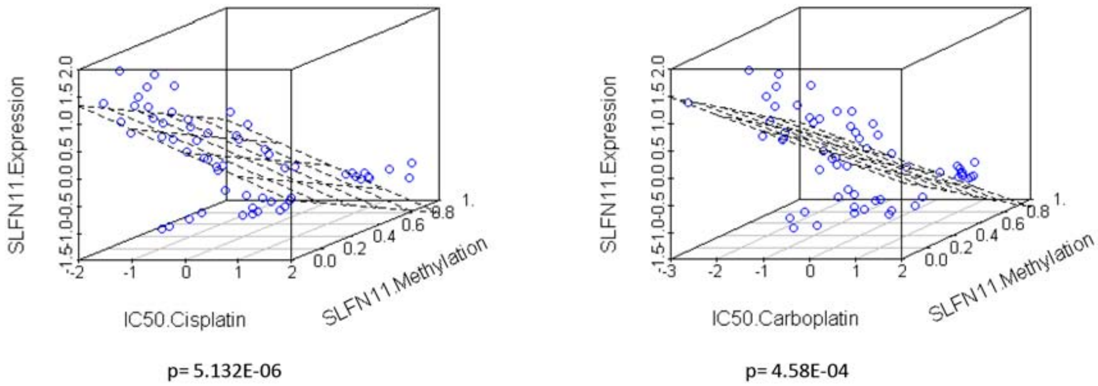

Supplementary Figure S2: Correlational study of SLFN11 methylation, expression and sensitivity to platinum drugs. Top, Pearson coefficients and p-values for SLFN11 promoter methylation average in relation with SLFN11 gene expression or IC50 values to platinum compounds along NCI-60 panel. Bottom, tridimentional dot-plot distribution of NCI-60 cell line according with their values of sensitivity to platinum drugs, SLFN11 expression and SLFN11 promoter methylation. Significance of common correlation between the 3 variables have been calculated by performing F-test and fitting the data to a linear model were methylation and expression data followed a bimodal distribution (‘low’ expression (<0) and ‘high’ expression (>=0), and ‘low’ methylation (<0.5) and ‘high’ methylation (>=0.5)).

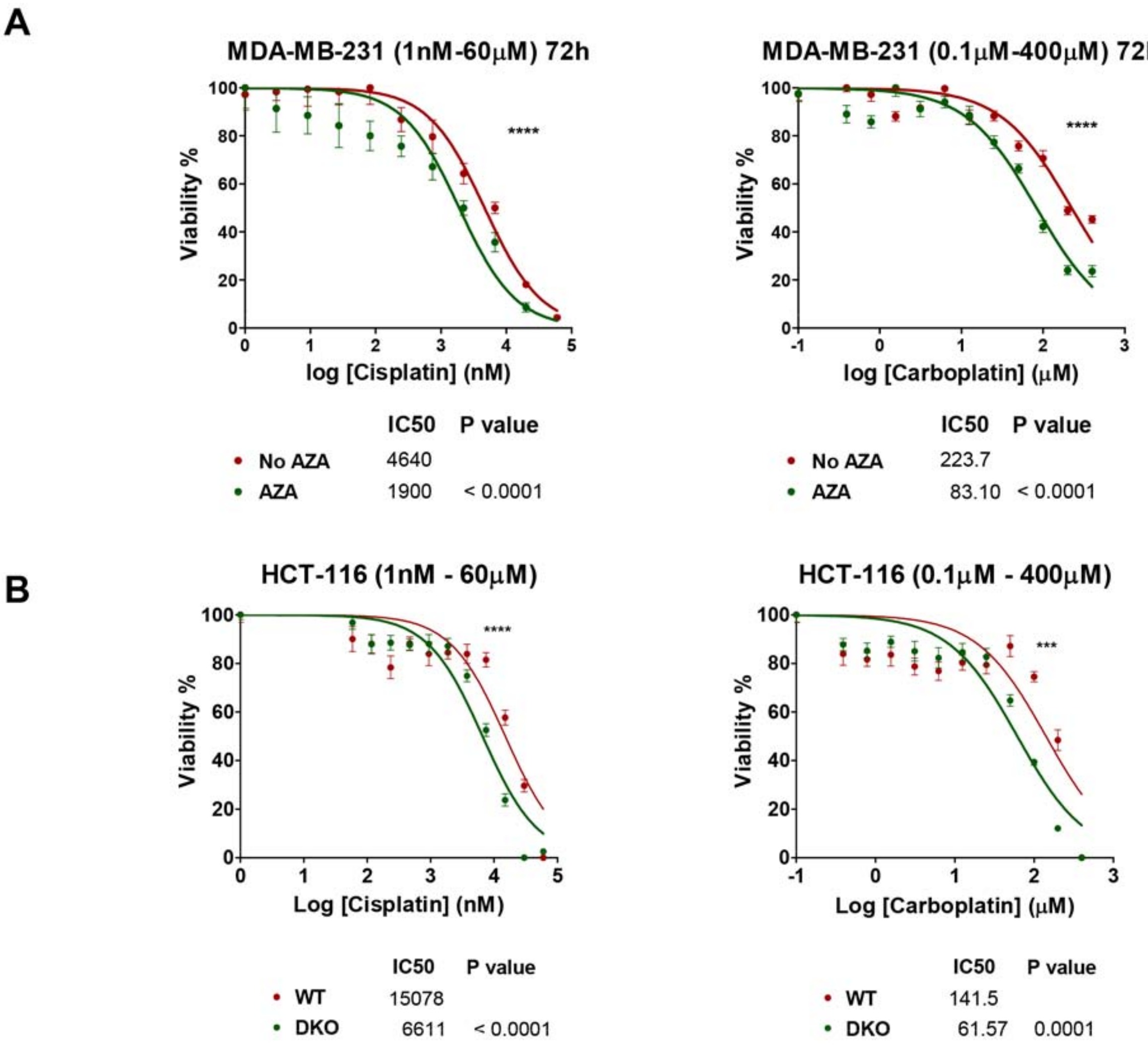

Supplementary Figure S3: DNA demethylation effect on sensitivity to platinum drugs in SLFN11 methylated cell lines. A. Cell viability (MTT assay) upon cisplatin and carboplatin use of the SLFN11 methylated cell line MDA-MB-231 treated with (AZA) or without (No AZA) the DNMT inhibitor azacytidine (72h). **B.** Comparison of cell viability (MTT assay) upon cisplatin and carboplatin use between the parental SLFN11 methylated HCT-116 cell line (WT) and the hypomethylated HCT-1116 DNMT-deficient isogenic cell line (DKO).

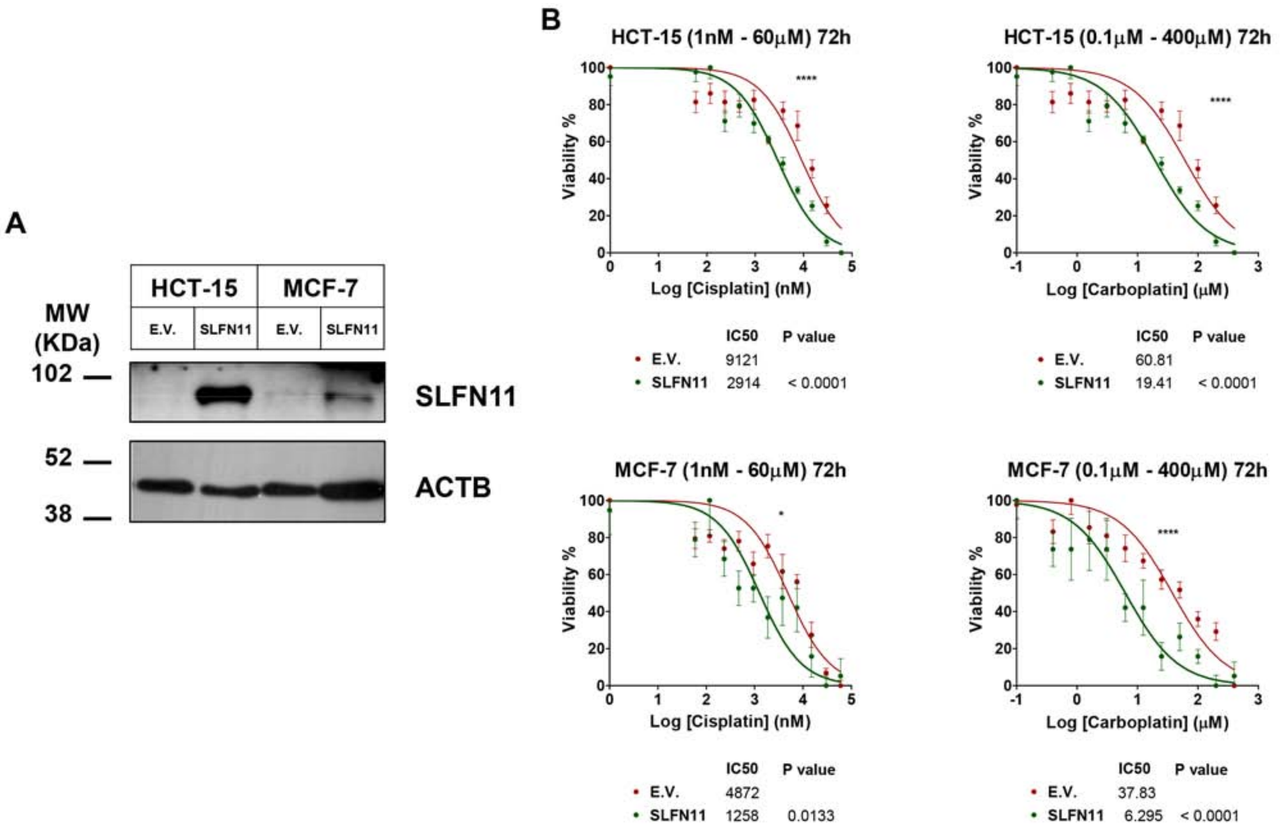

Supplementary Figure S4: Ectopic expression of SLFN11 in SLFN11 methylated cell lines (HCT-15 and MCF-7). A. Western blot showing the efficient re-expression of the SLFN11 protein using electroporation of the expression vector pcDNA4/TO carrying SLFN11 CDS in HCT-15 (colon) and MCF-7 (breast) cancer cell lines,. **B.** Cell viability determined by the 3-(4,5-dimethyl-2-thiazolyl)-2,5-diphenyl-2H-tetrazolium bromide (MTT) assay following exposure to cisplatin and carboplatin. pcDNA4/TO –SLFN11 over-expression gives rise to enhanced sensitivity to both drugs. The corresponding half-maximal inhibitory concentration (IC_{50}) values are also shown. E.V. stands for Empty vector.

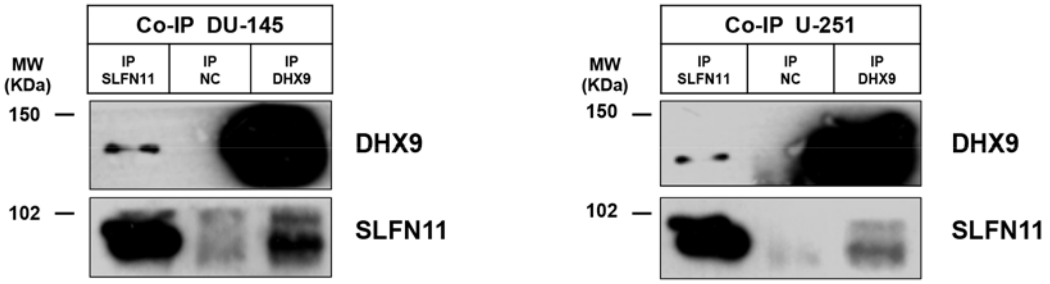

Supplementary Figure S5: Co-Immunoprecipitation of SLFN11 and DHX9 in DU-145 and U-251 cells. Western blot showing SLFN11 and DHX9 proteins in SLFN11 (IP SLFN11) and DHX9 immunoprecipitations (IP DHX9) of the SLFN11 promoter unmethylated cell lines DU-145 and U-251. Immunoprecipitation using antibody against nucleolin is used as a negative control (IP NC).
